# Effect of 2-Hydroxypropyl-*β*-cyclodextrin on Solubility of Sparingly Soluble Drug Derivatives of Anthranilic Acid

**DOI:** 10.3390/ijms12042383

**Published:** 2011-04-06

**Authors:** Urszula Domańska, Aleksandra Pelczarska, Aneta Pobudkowska

**Affiliations:** Department of Physical Chemistry, Faculty of Chemistry, Warsaw University of Technology, Noakowskiego 3, 00-664 Warsaw, Poland; E-Mails: apelczarska@ch.pw.edu.pl (A.P.); pobudka@ch.pw.edu.pl (A.P.)

**Keywords:** experimental solubility, 2-hydroxypropyl-*β*-cyclodextrin, solubility enhancement, thermodynamics of complex formation

## Abstract

Guest-host complex formation of three drug derivatives of anthranilic acid, mefenamic acid, niflumic acid, and flufenamic acid with 2-hydroxypropyl-*β*-cyclodextrin (2HP-*β*-CD) in aqueous solutions was investigated using “Phase solubility study” with UV-vis spectrophotometry. Solubility of sparingly soluble drugs has been improved by addition of 2HP-*β*-CD at two temperatures 298.15 K and 310.15 K and two pH values 2 and 7. The influence of different 2HP-*β*-CD concentration on solubility of drugs at different pH and temperatures has been investigated. The 2HP-*β*-CD-drug complex stability constants (*K*_s_), and dissociations constants (*K*_d_), as well as the thermodynamic parameters of reaction, *i.e.*, the free energy change (Δ*G*), the enthalpy change (Δ*H*) and the entropy change (Δ*S*), were determined. The experimental data indicated formation of 1:1 inclusion complexes, which were found effective binders increasing the solubility of drugs.

## 1. Introduction

Cyclodextrins (CDs), the natural oligosaccharides possessing a hydrophilic exterior and hydrophobic cavity, are well-known safe encapsulating materials [1–3]. In fact, new and promising CDs are continually being examined for their ability to improve complexity in order to obtain both higher solubility of drugs, and stabilizing or solubilizing agents [4–6]. The mechanism for this solubilization is rooted in the ability of CDs to form non-covalent dynamic inclusion complexes in solution. These CDs can serve as multi-functional drug carries, through the formation of these inclusion complexes, or the form of CD-drug conjugate, the ability to form non-inclusion based complexes, the formation of aggregates and related domains and the ability of CDs to form and stabilize supersaturated drug solutions and, thereby potentially serving as novel drug carriers. Thus, the investigation on inclusion of drug with CDs is of practical importance. A number of CD-based products have reached the market based on their ability to camouflage undesirable physicochemical properties. The hydroxypropyl derivatives of cyclodextrin are very often used in pharmacy due to their better complexation ability than that of natural cyclodextrin and they increase the solubility and bioavailability more than natural cyclodextrin [4,7–13]. Different CDs including *β*-CD were used to complex formation with candesartan cilexetil showing the highest constant of complex formation for *β*-CD with large enthalpy effect [8]. The 2-hydroxypropyl-*β*-cyclodextrin (2HP-*β*-CD) demonstrated more stable complexes than other *α*-, or *β*-, or *γ*- complexes of menadione [4]. The 2HP-*β*-CD was used to enhance the solubility of acyl ester prodrugs of ganciclovir in water [7]. The 2HP-*β*-CD was used for the complexation of triclosan, an antimicrobial agent with extremely low water solubility and it was proven that the solubility in aqueous ethanol showed 70-fold increase [10]. Glibenclamide has extremely poor aqueous solubility and resulting low bioavailability, thus to improve its capability the complexes with 2HP-*β*-CD were also presented [11]. The values of stability constants of flutamide-2HP-*β*-CD were very high in comparison with five other CDs [12] and lower for the complexation of ketoconazole-2HP-*β*-CD [13].

In the last years, several reviews describing the use of CDs in food processing have been published [14–16]. CDs may be used as a binding substance to flavors, fragrances, vitamins, as food additives [14]. CDs may be also recommended for applications in food technology to protect lipophylic food components that are sensitive to oxygen and light- or heat-induced degradation. CDs may be used as pharmaceuticals for controlled release of certain food constituents and to suppress unpleasant odors or tastes [15,16]. Again it was confirm that *β*-CD show higher binding constants than *α*-complexes [15].

Recently, the solubility of two drug derivatives of anthranilic acid (the mefenamic acid, MEF, and meclofenamic acid sodium salt, MASS) in three solvents—water, ethanol and 1-octanol—were investigated by us [17,18]. MEF is an anthranilic and indole acetic-type acid. It is a poorly soluble pharmaceutical, 0.17 × 10^−4^ mmol·dm^−3^ at *T* = 298.15 K [19] or 0.87 × 10^−4^ mmol·dm^−3^ at *T* = 298.15 K and pH between 3 and 6 [20]. An interesting analysis was provided for the solubility of MEF in water at *T* = 298.15 K influenced by the different excipients [20,21]. In conclusion it was stated that the mefanamic acid forms anionic dimers and trimers and excipients increase the solubility. The solubility of MEF in ethanol and 1-octanol at *T* = 298.15 K and pH 7 was 36.9 mmol·dm^−3^ and 13.07 mmol·dm^−3^, respectively [17]. A crystal structure of the inclusion complex of *β*-CD with MEF has already been determined by a combination of high-resolution synchrotron powder-diffraction data and molecular-mechanics calculations [22].

The chosen drugs, and especially FLU have been used in the treatment of arthritis and other illnesses related to muscular-skeletal problems [23]. The influence of pH on the optical properties of FLU in nanosystems was recently developed [24]. The influence of cyclodextrin and surfactants on micellar systems in aqueous solutions and the fluorescence of mixtures were described [25]. The solubility of FLU in water at *T* = 298.15 K is 2.38 × 10^−2^ mmol·dm^−3^ [19].

In this study we have focused our attention on the inclusion of drug derivatives of anthranilic acid with 2HP-*β*-CD, a steroidal molecule with cavity size suitable for hosting the large molecules. To understand the character and properties of the association, we investigated the effect of 2HP-*β*-CD on aqueous solubility and we evaluated the stoichiometry and stability of the complexes.

## 2. Results and Discussion

### 2.1. Solvent Effect and Solution Equilibrium

**Figure 1.**
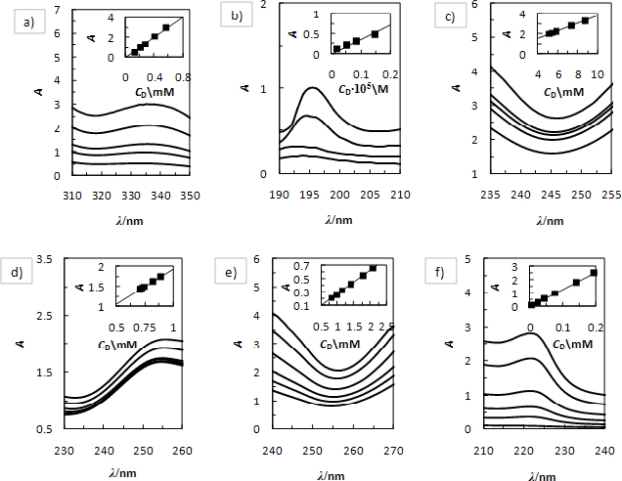
Absorption spectra of different concentrations of drugs at *T* = 298.15 K and their calibration curves. (**a**) Mefemamic acid, phosphate buffer I (pH 7.0); (**b**) Mefemamic acid, phosphate buffer II (pH 2.0); (**c**) Niflumic acid, phosphate buffer I (pH 7.0); (**d**) Niflumic acid, phosphate buffer II (pH 2.0); (**e**) Flufenamic acid, phosphate buffer I (pH 7.0); (**f**) Flufenamic acid, phosphate buffer II (pH 2.0).

The absorption spectra of drugs depend on pH of the solution due to protonation/deprotonation of nitrogen atom in the drug structure. Figure 1 presents examples of the absorption spectra of three drugs at different pHs.

The calibration curves are included in these figures. The absorption spectra were recorded in the range of 190–350 nm and for the calculation of 2HP-*β*-CD concentration the change of absorption was measured at 340 nm (pH 7.0), 195 nm (pH 2.0) for MEF; 245 nm (pH 7.0), and 250 nm (pH 2.0) for NIF and 255 nm (pH 7.0), and 225 nm (pH 2.0) for FLU, respectively.

**Figure 2.**
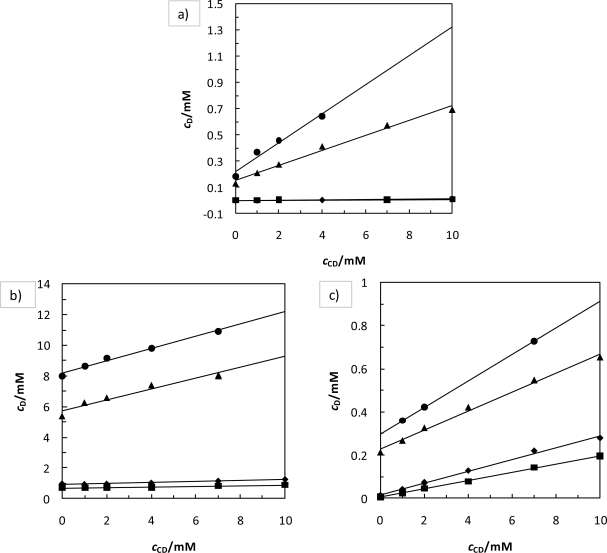
2HP-*β*-CD influence on the drug solubility at different temperatures and pHs. Experimental points: (▪) pH = 2 and *T* = 298.15 K; (♦) pH = 2 and *T* = 310.15 K; (▴) pH = 7 and *T* = 298.15 K; (•) pH = 7 and *T* = 310.15 K; Lines are drown to show the trends. (**a**) Mefenamic acid dependence; (**b**) Niflumic acid dependence; (**c**) Flufenamic acid dependence.

The aqueous solubility of the studied compounds were found to increase almost linearly with 2HP-*β*-CD concentration. The two exceptions are MEF and NIF at *T* = 298.15 K and pH = 2, for which the influence of 2HP-*β*-CD is linear but not very intensive (see Figure 2a and 2b). The solubility dramatically increases with an increase of pH and temperature (see Table 1).

Solubility enhance ratio, *R*, was calculated as a drug concentration at the highest cyclodextrin concentration with relation to drug concentration in the absence of cyclodextrin. Calculated values have been reported in Table 1. Solubility enhance ratio increases with an increase of temperature for MEF at pH = 2, 7, for NIF at pH = 2, and for FLU at pH = 2, 7 and slightly decreases with an increase of temperature for NIF at pH = 7. *R* decreases with an increase of pH for MEF and FLU, which demonstrates effects in drug release in the basic environment of intestinal part of the alimentary tract. The opposite effect is observed for NIF. The highest solubility enhance ratio, *R* is observed for FLU at pH = 2 (*R* = 31.0 at *T* = 298.15 K and *R* = 19.0 at *T* = 310.15 K) and MEF at pH = 2 (*R* = 17.3 at *T* = 310.15 K). In general, when the solubility of the drug is very low, the solubility enhance ratio, *R* is high. Substitution of hydroxyl groups surrounding the 2HP-*β*-CD rim by the hydroxypropyl group promotes the complex formation process for drug derivatives of anthranilic acid with polar carboxylic substituent. Perhaps the aromatic rings increase the 2HP-*β*-CD cavity dimensions and the carboxylic group stabilizing the outside hydrophilic groups and the binding of drug with 2HP-*β*-CD becomes more effective.

It was shown for MEF that the phenyl moieties were located outside the *β*-CD cavity, and the *N* atom that links the two phenyl rings is situated at the secondary face of the CD macrocycle with a hydrogen bond to the G4 glucose unit of the neighboring *β*-CD molecule [25].

### 2.2. Solvent Effect and Solution Equilibrium

Measurements of cyclodextrin-drug complex stability constants *K*_s_ and dissociation constants *K*_d_ are very important since these values indicate changes in physicochemical properties of a compound upon inclusion. Linear dependence of drug concentration to cyclodextrin concentration, with slope ratio below one usually assumes 1:1 ratio of complex.

The stability constant, *K*_s_, for complexes with 1:1 ratio is represented by:

(1) $$K_{\text{s}}=\frac{\left[\text{CD}\right]}{\left[\text{D}\right]\cdot \left[\text{C}\right]}$$

Where: [D] is pure drug concentration, [C] is pure cyclodextrin concentration, and [CD] is concentration of drug-cyclodextrin complexes in solution. Complex dissociation constant, *K*_d_, is represented by:

(2) $$K_{\text{d}}=\frac{1}{K_{\text{s}}}$$

For such systems, stability constant can be obtained by the following equation, investigated previously by Higuchi and Connors, 1965 [25]:

(3) $$K_{\text{s}}=K_{1:1}=\frac{\text{slope}}{c_{0}\cdot (1-\text{slope})}$$

Calculated values are reported in Table 1. The solubility data indicate that the largest enhancement of the solubility and the highest stability constant values were obtained for FLU at pH = 2 (*K*_s_ = 3055 M^−1^, *T* = 298.15 K; *K*_s_ = 1850 M^−1^, *T* = 310.15 K) and for MEF at pH = 2 (*K*_s_ = 1627 M^−1^, *T* = 310.15 K). These values suggest a relatively stable complex. The cavity of cyclodextrin exhibits a hydrophobic character, whereas the exterior is strongly hydrophilic. The process of complexation of MEF by 2HP-*β*-CD is high at two pHs and two temperatures, which can be connected with the association between the guest molecule and cyclodextrin molecule in solution via the two methyl groups of the MEF molecule stabilized by the carboxylic group outside the cyclodextrin. The structure of the three investigated compounds is similar. Two methyl groups in the molecule of MEF decrease the solubility in water in comparison with fluorine atoms of NIF and FLU and increase the complexation. The only difference between the NIF and FLU is the nitrogen atom in one of the aromatic rings in NIF. For these two substances, the molar mass is close to the same, and as a consequence of the additional nitrogen atom in NIF molecule, the stability constants, *K*s are lower than those for FLU at two pH and two temperatures. The significant differences in the stability constants for different drugs at various pHs and temperatures are connected to a different drug ionization degree, which increases with lowering pH, and with consequently less affinity for neutral 2HP-*β*-CD cavity. Data listed in Table 1 shows that at pH = 2, the FLU is almost fully ionized. A similar effect was observed for ketoconazole and flutamide with different CDs [13].

2HP-*β*-CD was found to be a more suitable complexating agent between four cyclodextrins for NIF, which was confirm by ^1^H NMR, UV-vis spectroscopy, densimetry and calorimetry (at pH = 7.4 and *T* = 298.15 K) [26]. The 1:1 stoichiometry of the 2HP-*β*-CD/NIF complexes was determined by Terekhova *et al*. [27], Zielenkiewicz *et al*. [26] and Bogdan *et al*. [28].

The phase solubility diagrams and stability constant was presented for FLU at pH = 6.75 and *T* = 298.15 K by Mura *et al.* [29].

Our value of stability constant for NIF at pH = 7 is log *K*_s_ = 1.98, whereas from the calorimetric, densimetry, UV-vis, and ^1^H NMR measurements at pH = 7.4 it is log *K*_s_ = 2.2, log *K*_s_ = 2.8, log *K*_s_ = 3.09 and log *K*_s_ = 2.3, respectively (*T* = 298.15 K) [26]. The literature value at pH = 12 is log *K*_s_ = 2.53 [28] and at pH = 7 log *K*_s_ = 2.72 [30] (*T* = 298.15 K). For FLU our value of stability constant at pH = 7 is log *K*_s_ = 2.30 and the literature value, obtained also from the phase-solubility diagrams at similar pH = 6.75 is log *K*_s_ = 2.36 [29] (*T* = 298.15 K). We can conclude at this moment that not only the temperature and pH but also the method of developing the stability constant is important.

Thermodynamic parameters of reactions *i.e.* the free energy change (Δ*G*), the enthalpy change (Δ*H*) and the entropy change (Δ*S*), can be obtained from the temperature dependence of stability constant [2]. Calculated values are reported in Table 2.

(4) $$\Delta G=-\text{R}T\text{ln}K_{1:1}$$

(5) $$\text{log}\left(\frac{K_{2}}{K_{1}}\right)=\frac{-\Delta H}{2.303\text{R}}\left(\frac{T_{2}-T_{1}}{T_{1}T_{2}}\right)$$

(6) ΔG=ΔH−TΔS

The thermodynamic parameters of the inclusion process estimated from the temperature dependence of the stability constants using van’t Hoff relation show different values of enthalpy, which is complexation parameter control. The highest value of enthalpy was observed for FLU (−4.9 kJ·mol^−1^) in comparison with the two other drugs (−10.0 kJ·mol^−1^ and −24.6 kJ·mol^−1^ for NIF and MEF, respectively). Negative values of Δ*H* and Δ*S* indicate that the van der Waals forces are responsible for the complexation process (observed only for MEF). In contrast, a huge hydrophobic interaction appears to contribute to the association of 2HP-*β*-CD and FLU, for which the entropy is high and positive (50.2 J·mol^−1^·K^−1^). The lower value of positive entropy for NIF indicates also hydrophobic interactions. The only difference between those two structures is aromatic nitrogen in the NIF structure, which increases hydrophobic interactions with 2HP-*β*-CD.

For the sake of comparison, only the free energy change (Δ*G*) of the reaction of complexation for NIF is similar to the literature value: ours is Δ*G* = −11.3 kJ·mol^−1^ (*T* = 298.15 K and pH = 7); the literature value is Δ*G* = −12.3 kJ·mol^−1^ (*T* = 298.15 K and pH = 7.4) [26]. The high negative value of enthalpy was obtained in previous work (−20.1 kJ·mol^−1^) and negative entropy [26]. These results were concluded from the densimetry and ^1^H NMR measurements.

## 3. Experimental Section

### 3.1. Materials and Instruments

Studied drugs were obtained from Sigma Aldrich, *i.e.,* mefenamic acid (CAS Registry No. 61-68-7; ≥0.99 mass fraction purity), niflumic acid (CAS Registry No. 4394-00-7; ≥0.99 mass fraction purity), flufenamic acid (CAS Registry No. 530-78-9; ≥0.99 mass fraction purity). The drugs were used without purification and were used as powder or small crystals. 2-Hydroxypropyl-*β*-cyclodextrin (2HP-*β*-CD) was delivered by ACROS Organics (CAS Registry No.128446-35-5; ≥0.97 mass fraction purity). Water used as a solvent was twice distilled, degassed and filtered with Milipore Elix 3. The buffers solution, were prepared from substances delivered by POCH, *i.e.*, potassium dihydrogen phosphate (CAS Registry No. 7758-11-4; ≥99.5% mass fraction purity), disodium hydrogen phosphate CAS Registry No. 7558-80-7; ≥99.5% mass fraction purity), hydrochloric acid (CAS Registry No. 7647-01-0; ≥35% mass fraction purity). All drugs were filtrated twice with Schott funnel with 4 μm pores. The names, abbreviations, structures and molar masses of the drugs and 2HP-*β*-CD are given in Table 3.

The UV-Vis spectrophotometer (Perkin–Elmer Life and Analytical Sciences, Shelton, USA) was used to determine the 2HP-*β*-CD influence on drug solubility. Maximum drug concentration was measured at different cyclodextrins concentrations, temperatures and pHs. Samples contained buffer solution with pH 7 or 2, 2HP-*β*-CD in different concentrations, *i.e*., 0, 1, 2, 4, 7 or 10 mmol·dm^−3^ and drug in excess. Samples were thermostated and mixed for 20 hours and then left for 4 hours for separation. The uncertainties of the temperature measurements were judged to be 0.1 K. Absorbance of each sample was measured with the UV-vis spectrophotometer.

### 3.2. Preparation of Solutions

The stability constants were determined by the measurements of the concentration of the drug in the sample as a function of the cyclodextrin concentration. Such a method is usually called “Phase solubility study” [31]. In this method complex stability constants are calculated from the obtained solubility data using a spectophotometric method. As a reference sample, the buffer + 2HP-*β*-CD solutions were used. Two buffers were prepared (mol concentration) *i.e.*, potassium dihydrogen phosphate (0.008), disodium hydrogen phosphate (0.008; buffer, pH = 7.0), potassium dihydrogen phosphate (0.05), hydrochloric acid (0.05, buffer, pH = 2.0). Samples were scanned with a scan step of 1 nm from 650 to 190 nm. Measurements at pH = 10 were not performed in all systems, because of the necessity of using too high drug concentrations and because of problems with separation of two phases.

## 4. Conclusions

The effect of 2HP-*β*-CD on the spectrophotometric behavior of three drugs, MEF, NIF and FLU was developed. The phase solubility diagram of the drugs in buffer aqueous solutions showed that the solubility of drugs increased linearly as a function of 2HP-*β*-CD concentration. The increase in solubility can be attributed to the formation of inclusion complexes between drug and 2HP-*β*-CD characterized by greater solubilities than that of drug alone. From the results it may be concluded that 2HP-*β*-CD forms 1:1 inclusion complexes with high stability and high stability constants, especially for FLU at pH = 2 and *T* = 298.15 K and *T* = 310.15 K as well as for MEF at pH = 2 and *T* = 310.15 K. The significant differences in stability constants at various pH values are connected with different drug ionization degree and various affinities for 2HP-*β*-CD cavity. The van der Waals forces coming from two methyl groups in MEF play an important role in the complex formation. Overall, the findings from this study strongly support the possibility of using 2HP-*β*-CD to significantly enhance the solubility of three drugs, mefenamic acid, niflumic acid and flufenamic acid in aqueous systems and enlarge the range of application of these anti-inflammatory pharmaceuticals.

## Figures and Tables

**Table 1.** The solubility (*C*) in water at two temperatures and two pHs (buffer solutions), calculated values of solubility enhance ratio (*R*), calculated values of cyclodextrin-drug complex stability constants *K*_s_ and dissociation constants *K*_d_.

**pH**	***T*/K**	***C*_D_/mM**	***R***	***K*_s_/M^−1^**	***K*_d_·10^3^/M**
**Mefenamic acid**					
2	298.15	0.001	6.4	539	1.86
	310.15	0.001	17.3	1627	0.62
7	298.15	0.155	4.6	383	2.61
	310.15	0.220	6.0	562	1.78
**Niflumic acid**					
2	298.15	0.698	1.3	26	38.9
	310.15	0.925	1.3	33	29.2
7	298.15	5.754	1.6	96	10.4
	310.15	8.171	1.5	82	12.2
**Flufenamic acid**					
2	298.15	0.006	31.0	3055	0.33
	310.15	0.015	19.0	1850	0.54
7	298.15	0.228	2.9	202	4.95
	310.15	0.298	3.0	218	4.59

**Table 2.** Thermodynamic parameters of reactions *i.e*., the free energy change (Δ*G*), the enthalpy change (Δ*H*) and the entropy change (Δ*S*) at *T* = 298.15 K and pH = 7 (buffer solution).

**Drug**	**Δ*G*/(kJ·mol^−1^)**	**Δ*H*/(kJ·mol^−1^)**	**Δ*S*/(J·mol^−1^·K^−1^)**
MEF	−14.7	−24.6	−33.0
NIF	−11.3	−10.0	4.3
FLU	−19.9	−4.9	50.2

**Table 3.** Investigated compounds: name, abbreviation, structure, and molar mass.

**Name of compound/abbreviation**	**Structural formula**	***M*/g · mol^−1^**
Mefenamic acid/MEF	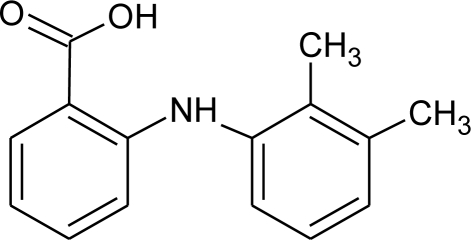	241.30
Niflumic acid/NIF	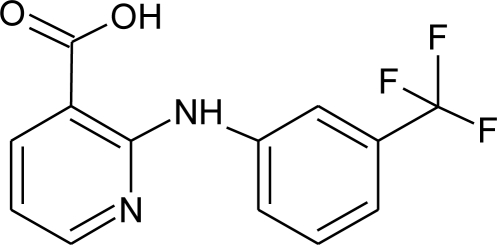	282.22
Flufenamic acid/FLU	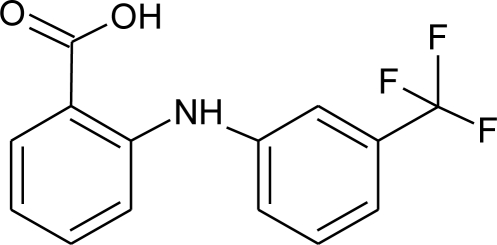	281.23
(2-Hydroxypropyl)-*β-*cyclodextrin	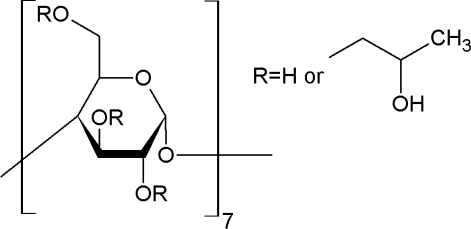	average ∼1.460
